# Relapse prevention with cariprazine: A focus on sex

**DOI:** 10.1192/j.eurpsy.2025.565

**Published:** 2025-08-26

**Authors:** C. Correll, Z. B. Dombi, P. Herman, Á. Barabássy

**Affiliations:** 1 The Donald and Barbara Zucker School of Medicine at Hofstra/Northwell, NY, United States; 2 Gedeon Richter Plc., Budapest, Hungary

## Abstract

**Introduction:**

Relapse refers to the recurrence of psychotic symptoms following a phase of improvement or stability. It often leads to the disruptive re-hospitalization of patients. Notably, a history of relapse is a strong indicator of future relapses and poorer outcomes. According to the literature, relapse rates might be higher in men compared to women due to better response to medication in women. Cariprazine (CAR), a D3-D2 partial agonist, has shown effectiveness in preventing relapse compared to a placebo in stabilized schizophrenia patients

**Objectives:**

We aimed to investigate whether there is a difference in the efficacy of CAR in preventing relapse by sex.

**Methods:**

A post-hoc analysis was conducted on data from a multicentre, randomized, double-blind, placebo-controlled, parallel-group study lasting approximately 96 weeks in adults with schizophrenia. The study included two phases: a 20-week open-label treatment phase and a double-blind treatment phase lasting up to 72 weeks. During the open-label phase, patients were stabilized on CAR at doses of 3.0-9.0 mg/day. Subsequently, they were randomized to either continue CAR (at fixed doses of 3.0, 6.0, or 9.0 mg/day) or switch to a placebo (PBO). Relapse was defined by a worsening of symptom scores on the Positive and Negative Syndrome Scale (PANSS), psychiatric hospital admission, aggressive behaviour, or suicide risk. In this analysis, patients were separately analysed based on their sex. Baseline characteristics, hazard ratios by sex during the double-blind phase were calculated.

**Results:**

Of 200 patients, 132 (66%) were male (M) and 68 (34%) were female (F). In the female group, 57% were receiving CAR treatment, while in the male group 47% were on CAR. The mean age of the patients was between 36-41 years. The open-label baseline PANSS scores were comparable. The adherence of patients during the double-label phase was similar in all four groups (98-99%).

More relapses were documented in the placebo groups (M: 47%, F: 48%) than in the CAR groups (M: 27%, F: 21%). In females, those who received CAR during the double-blind phase had 66% less risk for relapse (HR=0.34, 95%CI= 0.14-0.82) than those who were on placebo. Similarly, male patients on cariprazine had 49% less risk for relapse (HR=0.51, 95%CI= 0.28-0.91) than those receiving placebo. The Cox regression analysis between groups showed that sex of patients did not affect the risk of relapse significantly.

**Conclusions:**

In summary, sex does not seem to significantly influence risk of relapse. CAR decreases the risk of relapse compared to placebo in both males and females.

**Disclosure of Interest:**

C. Correll: None Declared, Z. Dombi Employee of: I am an employee of Gedeon Richter Plc., originator of cariprazine., P. Herman Employee of: I am an employee of Gedeon Richter Plc., originator of cariprazine., Á. Barabássy Employee of: I am an employee of Gedeon Richter Plc., originator of cariprazine.

